# Astaxanthin attenuates DNA fragmentation and cognitive decline in type 2 diabetic rats

**DOI:** 10.1038/s41598-025-04402-9

**Published:** 2025-06-20

**Authors:** Sohair A. Saleh, Gergess S. Hanna, Sobhy H. El-Naby, Suzy F. Ewida, Rasha S. A. Elseadawy, Basma S. Ghoneim, Shaimaa M. Motawea

**Affiliations:** 1 Clinical Physiology Department, Faculty of Medicine, Menoufia University, Shibin al Kawm, Egypt; 2 Genetics and Molecular Biology, Faculty of Science, Menoufia University, Shibin al Kawm, Egypt; 3 Basic Medical Science Department, Faculty of Medicine, New Mansoura University, Al Mansurah, Egypt; 4 Neurology Department, Faculty of Medicine, Menoufia University, Shibin al Kawm, Egypt

**Keywords:** Astaxanthin, Cognition, Hippocampus DNA, Phosphorylated tau, Diabetes type 2, Neuroscience, Endocrinology

## Abstract

Potential properties of astaxanthin include immunomodulation, antioxidant, and anti-inflammatory effects. In diabetic rats, we examined the potential impacts and underlying mechanisms of astaxanthin on hippocampal DNA, cognition, and glycemic status. Rats were divided into five equal groups: non-diabetic, and diabetic (non-treated, metformin treated, astaxanthin treated, and treated with a combination of metformin and astaxanthin). Both spatial and non-spatial memory and learning were assessed. IL-6, malondialdehyde, total antioxidant capacity, lipid profile, and glycemic status were assessed. Phosphorylated tau expression level was measured, and H&E section analysis was used to evaluate the hippocampal tissue. DNA fragmentation, intact DNA, and hippocampal RNA were evaluated. Induction of diabetes led to a reduction in cognitive abilities along with significant hyperglycemia, dyslipidemia, oxidative stress, hyperphosphorylation of tau, and DNA fragmentation. Astaxanthin as monotherapy or in combination with metformin improved cognitive functions with reduction of hyperglycemia, dyslipidemia, oxidative stress, hyperphosphorylation of tau, and DNA fragmentation.

## Introduction

The incidence of diabetes is rising globally. The number of individuals with diabetes is predicted to rise from 463 to 700 million by 2045 as a result of urbanization, obesity, and aging^1^.

Ninety to ninety-five percent of cases of diabetic mellitus “T2D” are caused by impaired insulin sensitivity in the target tissues, which causes a disruption in insulin secretion^2^. A new term, diabetes-associated cognitive decline “DACD”, has been proposed to support fields of research and aid in identifying the underlying causes of this condition^3^.

Insulin normally prevents tau phosphorylation and the formation of tau fibrils. Reduced levels of CSF insulin are linked to a higher amount of neurofibrillary tangle. Neurofibrillary tangles load is the best pathological marker of the degree of dementia in Alzheimer’s disease “AD”^4,5^.

Pro-inflammatory cytokines "IL-1β", “TNF”, and "IL-6" are found in higher concentrations in the brains of diabetics, and these cytokines are linked to memory impairment in the areas of spatial recognition^6^.

Oxidative stress has been shown to play a significant role in insulin resistance. Oxidative stress is caused by an excess of endogenous oxidative species, which damages cells and modifies signal pathways^7^. Reactive oxygen species “ROS” are produced in diabetes from a variety of sources, including both non-mitochondrial and mitochondrial sources^8^.

Antioxidants prevent free radicals from attaching to cells by stabilizing or neutralizing them. Humans have highly complex antioxidant systems (both enzymatic and non-enzymatic), which cooperate with one another to shield organ systems and cells from harm caused by free radicals. Antioxidants can be derived from external sources; incorporated into a diet or used as dietary supplements or synthesized internally^9^. The primary origin of commercial astaxanthin is the yeast Phaffia rhodozyma. Potential pharmacological properties of astaxanthin “Asx” include immunomodulation, antioxidants, anti-inflammatory, and anti-cancer effects. Astaxanthin exhibits strong antioxidant qualities because of its molecular makeup and arrangement in the plasma membrane, which promote the neutralization of reactive oxygen and nitrogen species. It also has strong anti-inflammatory effects that may be connected to these antioxidant effects. Possible advantages of astaxanthin for cardiovascular health was demonstrated by preclinical and clinical research^10^.

The first line as an anti-hyperglycemic medication for type 2 diabetes is the biguanide metformin “Met”. According to research, metformin exhibits strong insulin-sensitizing capabilities. As it may be able to pass through the blood–brain barrier, treatment with metformin may therefore ameliorate the cognitive impairment resulting from brain metabolic stress and neuroinflammation^11^.

The current study investigated the possible impact of the powerful antioxidant astaxanthin on the hippocampal DNA, glycemic status, and cognitive brain functions in rats with type 2 diabetes, as well as potential underlying processes. Additionally, using it in conjunction with other antidiabetic drugs as an adjuvant in the treatment of diabetes type 2.

## Material and methods

### Ethical approval

Our study used a randomized controlled animal experimental strategy, Faculty of Medicine, Menoufia University, Egypt and consented to by the national ethics committee with IRB No 19719PHYS73. The experiment followed the standards for Animal Research: Reporting of In Vivo Experiments (ARRIVE)^12^. G*Power was used for estimating sample size with power 80% and confidence interval 95%.

#### Experimental design

In this study, ninety adult male Wistar albino rats weighing between 150 and 200 g each were used. Ten rats were kept in 80 × 40 × 30 cm, properly ventilated cages at room temperature with a natural cycle of day and night and unrestricted access to water. Before beginning the trials, they were allowed to acclimate for 1 week by being fed a rat normal pellet diet “NPD” and tap water. Five equal experimental groups of eighteen animals each were created at random as: Non-Diabetic Group (ND), Diabetic Non-treated group (DNT), Diabetic metformin treated group (D + Met), Diabetic astaxanthin treated group (D + Asx), and Diabetic metformin and astaxanthin treated group (D + M + Asx).

For 4 weeks, rats of the ND group were fed “NPD”, in which fat represents 12% of the total calories^13^. They received a single injection of 0.1 ml of citrate buffer (streptozotocin “STZ” vehicle). Then they were given an oral daily dose of 1 ml of saline vehicle of metformin) and 1 ml of canola oil (a vehicle of astaxanthin) using oral gavage for 4 weeks. In the DNT group, type II diabetes was induced^13^, followed by offering rats free access to “HFD” for 4 weeks with daily oral administration of 1 ml of saline (a vehicle of metformin) and 1 ml of canola oil (a vehicle of astaxanthin) using oral gavage^14^. In the D + Met group, type II DM was induced, followed by daily oral administration of 200 mg/kg metformin (Sigma-Aldrich), dissolved in saline, and 1 ml of canola oil (a vehicle of astaxanthin) using oral gavage for 4 weeks^15^. These rats were fed “HFD” throughout the experimental period^14^. In the D + Asx group, Type II DM was induced, followed by daily oral administration of 1 mg/kg of astaxanthin (California Gold Nutrition, Madre Labs, LLC) dissolved in canola oil and 1 ml of saline (a vehicle of metformin) for 4 weeks using oral gavage^16^. These rats were fed a high-fat diet “HFD” throughout the experimental period^14^. In the D + M + Asx group, Type II DM was induced, followed by daily oral administration of 200 mg/kg metformin dissolved in saline and 1 mg/kg astaxanthin dissolved in canola oil for 4 weeks using oral gavage. These rats were fed “HFD” throughout the experimental period^14^.

At the end of the experimental period, rats of each group were randomly divided into 3 subgroups with 6 rats in each, and each subgroup was assessed for cognition by one of the following tests:

- Subgroup-a was tested for spatial memory and learning using the Morris water maze test.
- Subgroup-b was tested for non-spatial memory using the novel object recognition test.
- Subgroup-c was tested for spatial working (short-term) memory using the Y-Maze spontaneous alteration test.

### Biochemical assessment

Blood and serum samples were collected to measure the glycaemic state, including HbA1c (Product Code: 51835001, Large, Riomidi, France), fasting serum glucose (Sigma-Aldrich, St. Louis, MO, USA), and serum insulin (Sigma-Aldrich, St. Louis, MO, USA). HOMA-IR = (insulin × glucose)/405; for glycemia in mg/dL was calculated for the assessment of insulin resistance^17^. Lipid profile: total cholesterol (Biodiagnostic Company, Cairo, Egypt), triglycerides (Biodiagnostic Company, Cairo, Egypt), high-density lipoprotein “HDLc” (Biodiagnostic Company, Cairo, Egypt), and low-density lipoprotein “LDLc” were calculated according to Friedwald’s formula^18^. Serum malondialdehyde “MDA” (Biodiagnostic Company, Cairo, Egypt), total antioxidant capacity (Biodiagnostic Company,Cairo, Egypt), and interleukin-6 "IL-6" (Catalogue Number: EI1006-1, AssayMax ™ ELISA Kits, Assay Pro. Company, U.S.A.). Measurements were performed according to the manufacturer’s protocols for each kit.

### Rat model with type II diabetes

Rats were fed “HFD” (Table1)^13^ consisting of 17% carbohydrates, 25% protein, and 58% fat as a percentage of total calories for 2 weeks in order to cause type 2 diabetes. Rats were given a single intraperitoneal injection of 35 mg/kg of “STZ” dissolved in citrate buffer (pH 4.4) after they had fasted for 12 h^19^. In order to avoid hypoglycemia, a 5% glucose solution was then administered.

**Table 1:** Constituents of the HFD.

Ingredients	Diet (g/kg)
Powdered NPD (Egyptian market)	365
Lard (Egyptian market)	310
Casein (Difco, Becton Dickinson, France)	250
Cholesterol (Sigma–Aldrich, Cairo, Egypt)	10
Vitamin and mineral mix (Sigma–Aldrich, Cairo, Egypt)	60
dl-Methionine (Sigma–Aldrich, Cairo, Egypt)	0.3
Yeast powder (Egyptian market)	1
Sodium chloride (Egyptian market)	1

By utilizing a glucometer (ACCU-CHEK) and tail vein puncture to measure fasting blood glucose levels, diabetes mellitus was confirmed 72 h after the injection. Rats classified as diabetic^19^ were limited to those with fasting blood glucose levels ≥ 150 mg/dl during selection. “HFD” was given to these rats for the duration of the investigation^14^.

### Memory and cognition assessment

#### Morris water maze test (MWM)^20^

Several extra-maze cues around a circular pool made of stainless steel. There were two main axes assigned for the maze, and each line that divided it perpendicularly to the other created an imagined " + ". West "W", North "N", South "S", and East "E" are the four cardinal points that are marked at the end of each line. Four equal quadrants are produced by dividing the maze in this fashion. Midway between the center and the wall, the platform is placed in the middle of the “SW” quadrant for every trial. Using a semi-random start position set, rats were trained for four trials per day for three days. If a rat could not locate the platform in sixty seconds, it was then manually guided to the platform and remained there for almost fifteen seconds. Following each trial, the rats were permitted to rest on the platform for approximately thirty seconds (inter-trial time). We calculated the rats’ escape latency in seconds needed to get to the platform for each trial. A probe (transfer) experiment was administered 24 h following the final acquisition day in order to evaluate reference memory at the conclusion of learning.

#### Novel object recognition test^21^

An open-field device with dimensions of 40 cm in height, 50 cm in width, and 50 cm in length. We made sure that rats could climb on both of the rat-sized things. Three replicas of the familiar object were given—two for training and one for testing—in an effort to lessen induced preference. The items were completely cleaned (70% ethanol) both before and after usage and were constructed of non-breakable material. For three days, we put each rat through a novel item recognition test, which consisted of three phases: a habituation phase (10 min for 1 day); a training phase (5 min for 1 day); and a test phase (5 min for 1 day). The discrimination index and recognition index are used to measure rats’ cognitive abilities.

- Discrimination index = [(novel object exploration time − familial object exploration time)/total exploration time × 100%].
- Recognition index = [(novel object exploration time/total exploration time) × 100%].

#### Y-maze test^22^

Unpainted wood made up the maze. The arms measured 40 cm in length, 11 cm in height, and 12 cm in width. They were symmetrically spaced at a 120° angle. At its longest length of 15 cm, the arm converged in an equilateral triangular core region. The maze’s arms are designated as either arm A, arm B, or arm C. Rats were housed in the room for thirty minutes before the test to let them become used to it. One rodent was positioned in arm B, the arm nearest the researcher, near the middle of the Y-Maze. For eight minutes, each rodent was free to explore the Y-maze. The rodent’s current location is recorded each time it enters a new arm. Calculate spatial cognition as measured by spontaneous alternation: the number of successful alternations/(the total number of entries − 2) × 100.

#### Collection of blood samples^23^

Retro-orbital blood samples (three milliliters) from each rat were obtained after an overnight fast. The blood was collected in two clean graduated tubes; one tube had EDTA added for measuring HbA1C, while the other tube was left plain for clotting, centrifuged, and the supernatant serum was collected. The serum was then stored at -80 degrees until it was needed.

#### Brain extraction and isolation of hippocampus^24^

Rats were killed via cervical elongation and dislocation, and their brains were removed, split in two halves, so the hippocampal region could be seen. Using two Dumont No. 5 forceps, the right hippocampus’ tissue was removed by exposing the ventral side of the brain and removing the midbrain. The specimen was then preserved at -80 degrees to allow for the extraction of DNA and RNA and the measurement of its flow rate^25,26^.

### Histopathological examinations

After being paraffin embedded and treated in 4% paraformaldehyde, the left hippocampal tissue was ready for H&E staining and X200 magnification light microscope inspection. A monoclonal antibody against phosphorylated tau was incubated, and immunohistochemistry was used to quantify the amount of phosphorylated tau expression in the sections that had already been produced. Sections were seen at X400 magnification using a light microscope, and the immunoreactive score “IRS” for each group was determined^27^.

### Statistical analysis

Mean ± standard deviation of the mean "S.D." was used to express the data. The statistical package for social sciences “SPSS”, version 20, for Windows, was used to analyze the data (SPSS Inc., Chicago, Illinois, USA). Tukey’s multiple comparison tests were used to assess the difference between different groups for the parametric parameters, and one-way analysis of variance (ANOVA) was used to determine statistical significance, which was defined as *P* < 0.05 for all experiments.

Within the experimental groups, correlation analysis was used to determine the degree of association between pathological alterations and biochemical and cognitive brain functioning. At the 0.05 level, the correlation was significant.

## Results

The mean values of fasting serum glucose, Hb A1C and HOMA-IR of DNT group were significantly higher, while the mean value of fasting serum insulin was significantly lower when compared to the corresponding values of ND group.

The mean values of fasting serum glucose, Hb A1C and HOMA-IR of D + Met and D + Asx treated groups were significantly lower when compared to DNT group, while remained significantly higher when compared to ND. The mean values of fasting serum insulin levels of D + Met and D + Asx treated groups were significantly higher when compared to DNT group and remained significantly lower when compared to ND. The mean values of fasting serum glucose, Hb A1C and HOMA-IR of D + Asx treated group were significantly higher while the mean value of fasting serum insulin was substantially lower when compared to D + Met treated group (Table 2).

**Table 2:** Glycemic state [fasting serum glucose (mg/dl), Hb A1C (% of normal) and insulin (µIU/ml) and HOMA-IR].

	ND	DNT	D + Met	D + Asx	D + M + Asx
Fasting serum glucose (mg/dl)	87.2 ± 13.57	426.7 ± 183.05*	137.2 ± 27.37*@	210.3 ± 27.41*@$	105.25 ± 9.57@$¥
Hb A1C (% of normal)	2.1 ± 0.29	6.8 ± 0.34*	3.6 ± 0.28*@	4.3 ± 0.19*@$	2.8 ± 0.24@$¥
Fasting serum insulin (µIU/ml)	11.8 ± 0.63	6.3 ± 0.34*	9.8 ± 0.55*@	7.8 ± 0.90*@$	10.6 ± 0.62@$¥
HOMA-IR	1.9 ± 0.17	6.6 ± 2.8*	3.1 ± 0.64*@	4.2 ± 0.59*@$	2.1 ± 0.26@$¥

#No. of rats in each group was 18. Results are represented as mean ± (S.D.). *, @, $ and ¥ beside values indicate that the values are significantly different, when compared with the corresponding values of C, DNT, D + Met and D + Asx group respectively.

Fasting serum glucose level, Hb A1C and HOMA-IR of D + M + Asx treated group were noticeably lower, while the mean value of fasting serum insulin level was substantially higher when compared to DNT group, whereas revealed insignificant changes when compared to ND. The mean values of fasting serum glucose, Hb A1C and HOMA-IR of D + M + Asx treated group were significantly lower when compared to D + Met and D + Asx treated groups, while the fasting serum insulin was significantly increased when compared to D + Met and D + Asx treated groups (Table2).

The mean value of fasting serum total cholesterol, Triglycerides and LDLc levels of DNT group were higher while the mean value of fasting serum level of HDLc was considerably lower when compared to ND group.

**Fig. 1:**
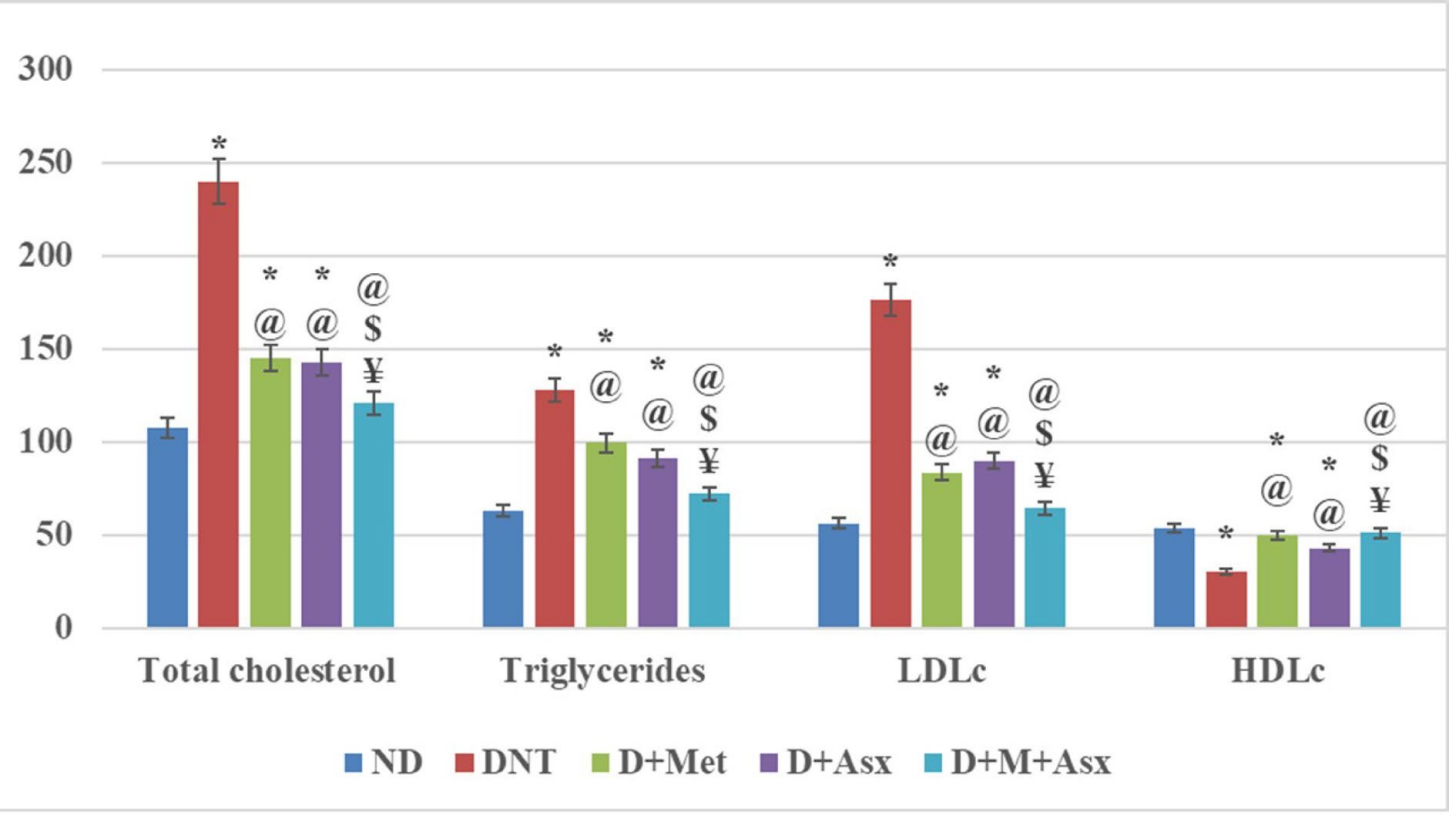
Total cholesterol (mg/dl), Triglycerides (mg/dl), LDLc (mg/dl) and HDLc (mg/dl) in non-diabetic (ND), diabetic non-treated (DNT), diabetic metformin-treated (D + Met), diabetic astaxanthin-treated (D + Asx) and diabetic combined metformin and astaxanthin treated groups (D + M + Asx). [*significant when compared to ND group, #significant when compared to DNT group, $ significant when compared to (D + Met), ¥ significant when compared to(D + Asx)]. Data are shown as means + SD (n = 10). ANOVA was used to make group comparisons; Significance = *P* < 0.05). # No. of rats in each group was 18.

The mean values of fasting serum total cholesterol, triglycerides and LDLc of D + Met were significantly less than those of the DNT group, while remained significantly higher in contrast to ND. The mean value of fasting serum HDLc was significantly higher in D + Met when compared to DNT group, while staying significantly lower when compared to ND (Fig. 1).

The fasting total cholesterol, triglycerides and LDLc of D + Asx treated group were significantly lower when compared to DNT group, while remained significantly higher when compared to ND group, with insignificant changes when compared to D + Met treated group. The mean values of fasting serum HDLc of D + Asx treated group was significantly higher when compared to DNT group, while remained significantly lower in comparison to the ND group and showed insignificant changes in contrast to D + Met treated group (Fig. 1).

The fasting total cholesterol, triglycerides and LDLc of D + M + Asx treated group were significantly lower in comparison to DNT group, with insignificant changes in all parameters when compared to ND group. The mean values of fasting serum total cholesterol, Triglycerides and “LDLc” of D + M + Asx treated group were significantly diminished when compared to D + Met treated and D + Asx treated groups, while fasting serum “HDLc” was significantly elevated in comparison to D + Met and D + Asx treated groups (Fig. 1).

The mean value of fasting serum "IL-6" of DNT was significantly elevated when compared to ND group. The mean values of fasting serum "IL-6" of D + Met and D + Asx treated groups were markedly reduced when compared to DNT group, while remained significantly higher when compared to ND group. The mean value of fasting serum "IL-6" of D + Asx treated group was insignificantly changed when compared to D + Met treated group. The mean value of fasting serum "IL-6" of D + M + Asx treated group was significantly lower in comparison to D + Met and D + Asx treated groups, while showed insignificant change when compared to ND group (Table 3).

**Table 3:** Serum IL-6 (pg/ml), Total Antioxidant capacity (mMol/L) and Lipid peroxidase (nMol/ml).

	ND	DNT	D + Met	D + Asx	D + Met + Asx
IL-6 (pg/ml)	22.1 ± 4.26	81.78 ± 6.23*	41 ± 3*@	41.68 ± 5.66*@	27.14 ± 2.34@$¥
Total Antioxidant capacity (mMol/L)	0.9 ± 0.05	0.3 ± 0.03*	0.6 ± 0.05*@	0.75 ± 0.06*@$	0.9 ± 0.05@$¥
Lipid peroxidase (nMol/ml)	2.0 ± 2.6	4.93 ± 7.2*	3.58 ± 4.4*@	2.72 ± 5.8*@$	2.35 ± 7.5@$

#No. of rats in each group was 18. Results are represented as mean ± (S.D.). *, @, $ and ¥ beside values indicate that the values are significantly different, when compared with the corresponding values of C, DNT, D + Met and D + Asx group respectively.

The mean value of fasting serum “TAC” of DNT group was significantly lower, while fasting serum lipid peroxidase activity was significantly higher when compared to ND group. The mean value of fasting serum “TAC” of D + Met treated group was significantly increased compared to DNT group, while stayed significantly lower when compared to ND group. Fasting serum lipid peroxidase activity was considerably reduced when compared to DNT group, while remained elevated when compared to ND group (Table 3).

The mean value of fasting serum “TAC” of D + Asx treated group was significantly increased in comparison to DNT group, even though stayed significantly lower when compared to ND group, while fasting serum lipid peroxidase activity of D + Asx treated group was significantly reduced when compared to DNT group, while remained significantly elevated when compared to ND group. The mean value of fasting serum “TAC” of D + Asx treated group was significantly elevated compared to D + Met treated group, while fasting serum lipid peroxidase activity was significantly diminished when compared to D + Met treated group (Table 3).

The mean value of fasting serum “TAC” of D + M + Asx treated group was significantly higher when compared to DNT group, while fasting serum lipid peroxidase activity was significantly lower when compared to DNT group, while D + M + Asx treated group showed insignificant changes in both parameters when compared to ND group. The mean value of fasting serum TAC of D + M + Asx treated group was higher when compared to D + Met and D + Asx treated groups , while fast serum lipid peroxidase activity was considerably lower when compared to D + Met treated group and showed insignificant change in comparison to D + Asx treated group (Table 3).

The escape latency of the probe trial of “MWM” test of DNT group was significantly elevated in comparison to ND group. The escape latency of the probe trial of MWM test of D + Met and D + Asx treated groups were significantly lower when compared to DNT group, while continuing to be significantly greater than the ND group. The escape latency of the probe trial of MWM test of D + Asx treated group showed insignificant change when compared to D + Met treated group. The escape latency of the probe trial of MWM test D + M + Asx treated group was significantly lower in comparison to DNT group, while showed insignificant change when compared to ND group. The escape latency the probe trial of “MWM” test of D + M + Asx treated group was significantly lower in comparison to D + Met and D + Asx treated groups (Table 4).

**Table 4:** Cognitive functions [escape latency of Morris water maze, discrimination index (d2) and recognition Index (d3) of NOR test and alterations of Y-maze].

	ND	DNT	D + Met	D + Asx	D + M + Asx
Morris water maize; Escape latency of probe trial (in seconds) (Subgroup-a: 6 rats)	4.8 ± 1.30	12.8 ± 5.99*	8.17 ± 1.47*@	7.8 ± 0.84*@	5.23 ± 1.60@$¥
NOR—d2 (Subgroup-b: 6 rats)	0.35 ± 0.25	− 0.09 ± 0.23*	0.14 ± 0.15*@	0.17 ± 0.25*@	0.28 ± 0.13@$¥
NOR—d3 (Subgroup-b: 6 rats)	67.55 ± 12.35	35.56 ± 11.75*	51.23 ± 7.54*@	53.47 ± 12.75*@	65.96 ± 6.47@$¥
Y- maze (% of total entries) (Subgroup-c:6 rats)	71.43 ± 16.24	31.0 ± 7.01*	49.2 ± 12.33*@	47.65 ± 8.91*@	62.5 ± 4.87@$¥

#No. of rats in each group was 18. Results are represented as mean ± (S.D.). *, @, $ and ¥ beside values indicate that the values are significantly different, when compared with the corresponding values of C, DNT, D + Met and D + Asx group respectively.

The mean values of discrimination index (d2) and recognition index (d3) of DNT group were significantly lower when compared to ND group. The mean values of (d2) and (d3) of D + Met were significantly greater when compared to DNT group, while remained significantly lower when compared to ND group.

The mean values of (d2) and (d3) of D + Asx treated group were significantly higher when compared to DNT group, while staying significantly lower when compared to ND group, D + Asx treated group showed insignificant changes in both indeces when compared to D + Met treated group (Table 4).

The mean values of (d2) and (d3) D + M + Asx treated group were significantly higher when compared to DNT group, while showed insignificant changes when compared to ND group. The mean values of (d2) and (d3) of D + M + Asx treated group were significantly higher when compared to D + Met treated group, and also were significantly higher in comparison to D + Asx treated group (Table 4).

The mean value of % of spontaneous alternation of Y-maze of DNT group were significantly lower when compared to ND group. The mean value of % of spontaneous alternation of Y-maze of D + Met and D + Asx treated groups were significantly higher when compared to DNT group, while remained substantially lesser in comparison to ND group. The mean value of % of spontaneous alternation of Y-maze of D + Asx treated showed insignificant change when compared to D + Met treated group. The mean value of % of spontaneous alternation of Y-maze of D + M + Asx treated group was significantly higher in comparison to DNT group, while showed insignificant change when compared to ND group. The mean value of % of spontaneous alternation of Y-maze of D + M + Asx treated group was significantly greater in comparison to D + Met and D + Asx treated groups (Table 4).

**Fig. 2:**
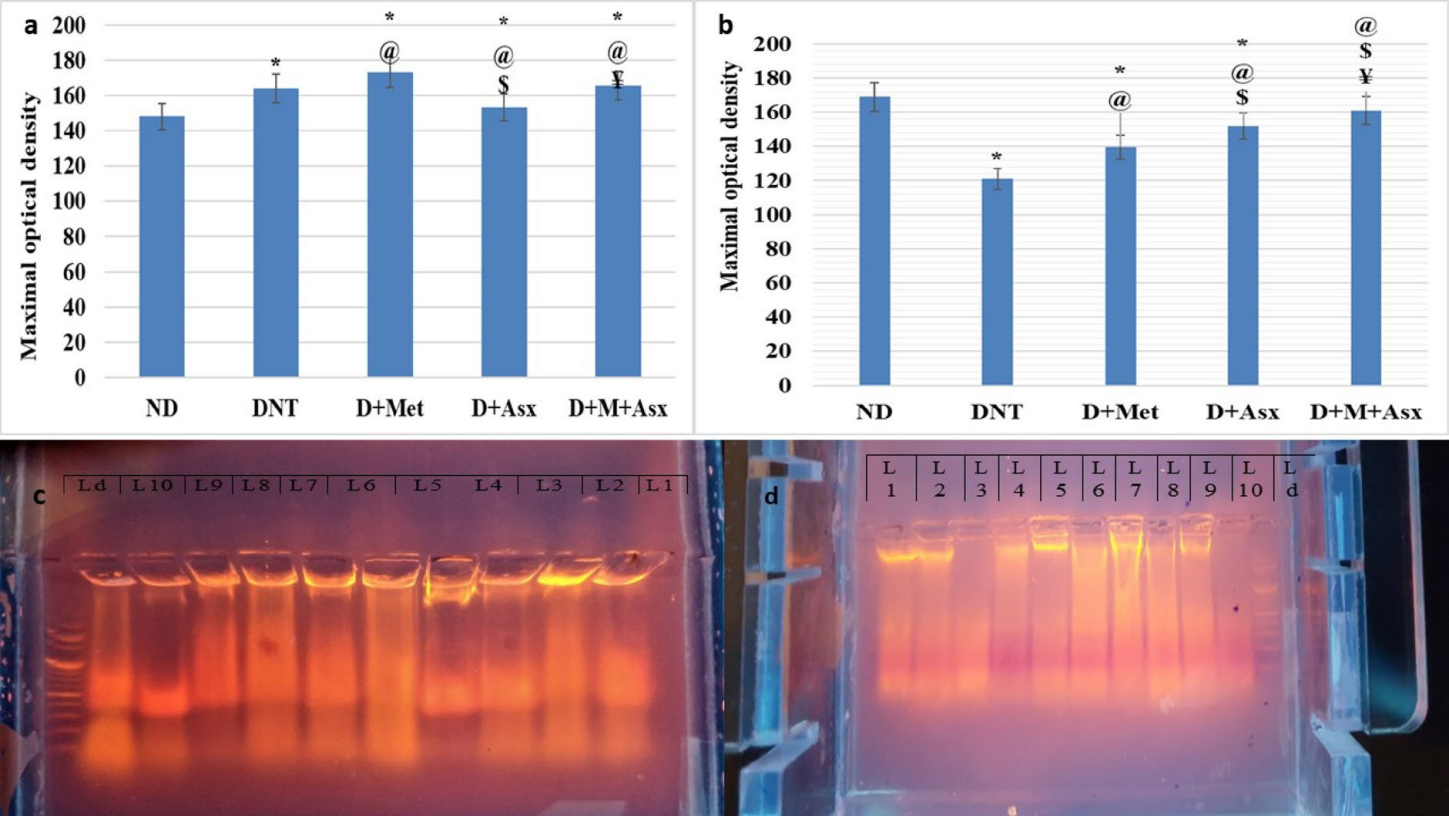
(**a**) Maximal optical density of Hippocampal RNA and (**b**) Maximal optical density of intact hippocampal DNA in non-diabetic (ND), diabetic non-treated (DNT), diabetic metformin-treated (D + Met), diabetic astaxanthin-treated (D + Asx) and diabetic combined metformin and astaxanthin treated groups (D + M + Asx) [*significant when compared to ND group, #significant when compared to DNT group, $ significant when compared to (D + Met), ¥ significant when compared to(D + Asx)]. Data are shown as means + SD (n = 10). ANOVA was used to make group comparisons; Significance = *P* < 0.05). (**c**) Digital photograph of electrophoretic pattern of RNA of rat hippocampal tissue lysate. (**d**) Digital photograph of electrophoretic pattern of DNA of rat hippocampal tissue lysate, Lane 1 & 2: ND group, Lane 3 & 4: DNT, Lane 5 & 6: D + Met, Lane 7 & 8: D + Asx, Lane 9 & 10: D + M + Asx. Lane d: DNA laddar # The number of rats in each group was 18. # No. of rats in each group was 18.

The mean value of maximal optical density of hippocampal RNA of DNT treated group was significantly higher when compared to ND group. The mean values of maximal optical density of hippocampal RNA of D + Met treated group were significantly higher when compared to ND and DNT treated group (Fig. 2a).

The mean value of maximal optical density of hippocampal RNA of D + Asx treated group was significantly lower in comparison to DNT group, while remained significantly higher when compared to ND group. The mean values of maximal optical density of hippocampal RNA of D + Asx treated group was significantly lower when compared to D + Met treated group (Fig. 2a).

The mean values of maximal optical density of hippocampal RNA of D + M + Asx treated group were significantly higher when compared to ND and DNT treated group. The mean value of maximal optical density of hippocampal RNA of D + M + Asx treated group was significantly higher when compared to D + Asx treated group, while showed insignificant change when compared to D + Met treated group (Fig. 2a).

The mean value of maximal optical density of intact hippocampal DNA of DNT group was significantly lower when compared to ND group. The mean values of maximal optical density of intact hippocampal DNA of D + Met and D + Asx treated groups were significantly lower, while D + M + Asx treated group showed insignificant change when compared to ND group. The mean values of maximal optical density of intact hippocampal DNA of D + Met, D + Asx and D + M + Asx treated groups were significantly higher when compared to DNT group. The mean values of maximal optical density of intact hippocampal DNA of D + Asx and D + M + Asx treated groups were significantly higher when compared to D + Met treated group (Fig. 2b).

The mean value of maximal optical density of intact hippocampal DNA of D + M + Asx treated was significantly higher when compared to D + Asx treated group (Fig. 2b).

**Fig. 3:**
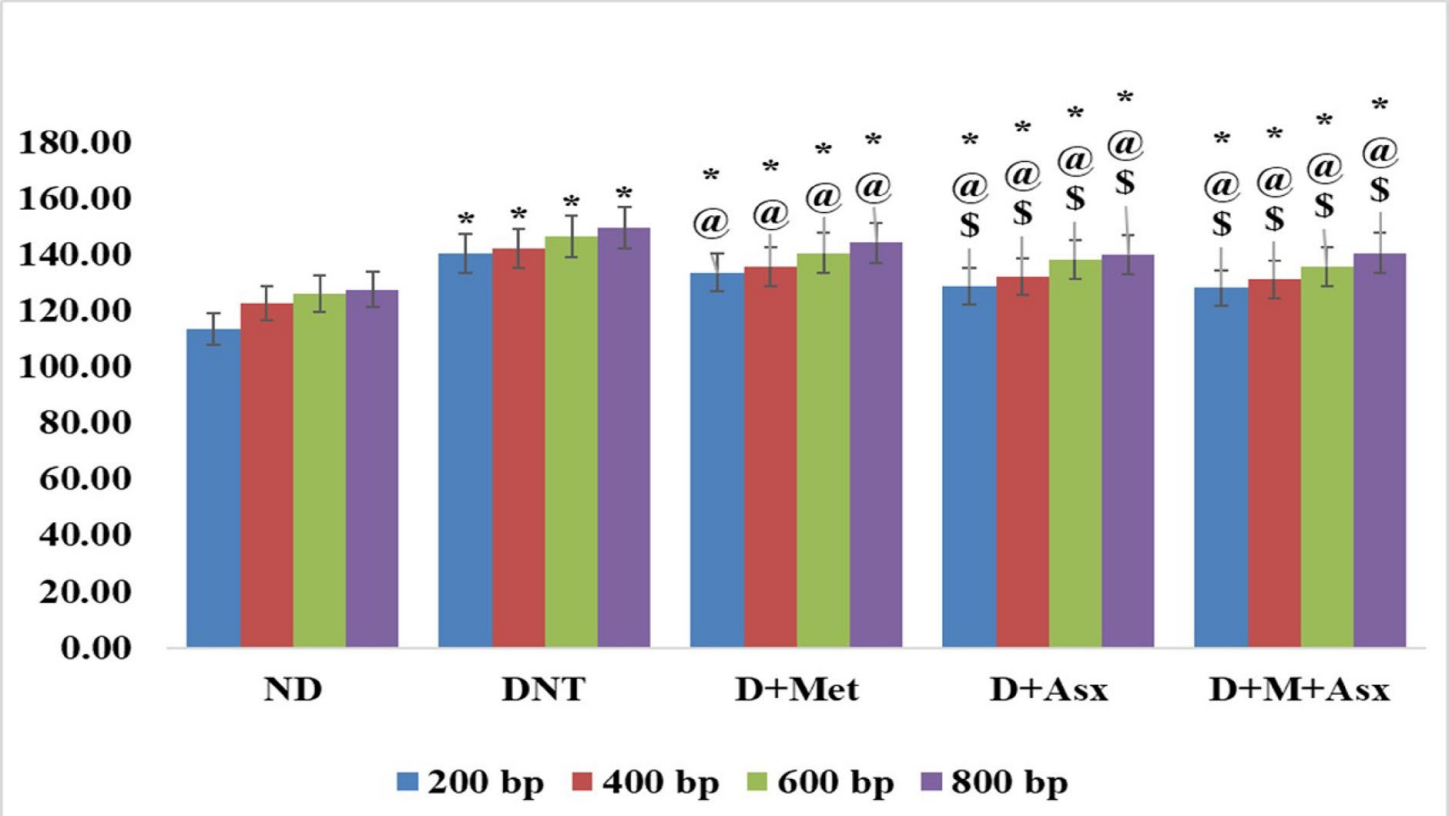
Maximal optical density of apoptotic fragments of DNA in non-diabetic (ND), diabetic non-treated (DNT), diabetic metformin-treated (D + Met), diabetic astaxanthin-treated (D + Asx) and diabetic combined metformin and astaxanthin treated groups (D + M + Asx). [*significant when compared to ND group, #significant when compared to DNT group, $ significant when compared to (D + Met), ¥ significant when compared to (D + Asx)]. Data are shown as means + SD (n = 10). ANOVA was used to make group comparisons; Significance = *P* < 0.05). # No. of rats in each group was 18.

The mean value of maximal optical density of apoptotic fragments of DNA of DNT group was significantly greater in comparison to ND group. The mean values of maximal optical density of apoptotic fragments of DNA of D + Met, D + Asx and D + M + Asx treated groups were significantly lower than those of DNT group while remained considerably greater when compared to ND group. The mean values of maximal optical density of apoptotic fragments of DNA of D + Asx and D + M + Asx treated groups were significantly lower when compared to D + Met treated group, while the mean value of maximal optical density of apoptotic fragments of DNA of D + M + Asx treated was insignificant when compared to D + Asx treated group (Fig. 3).

**Fig. 4:**
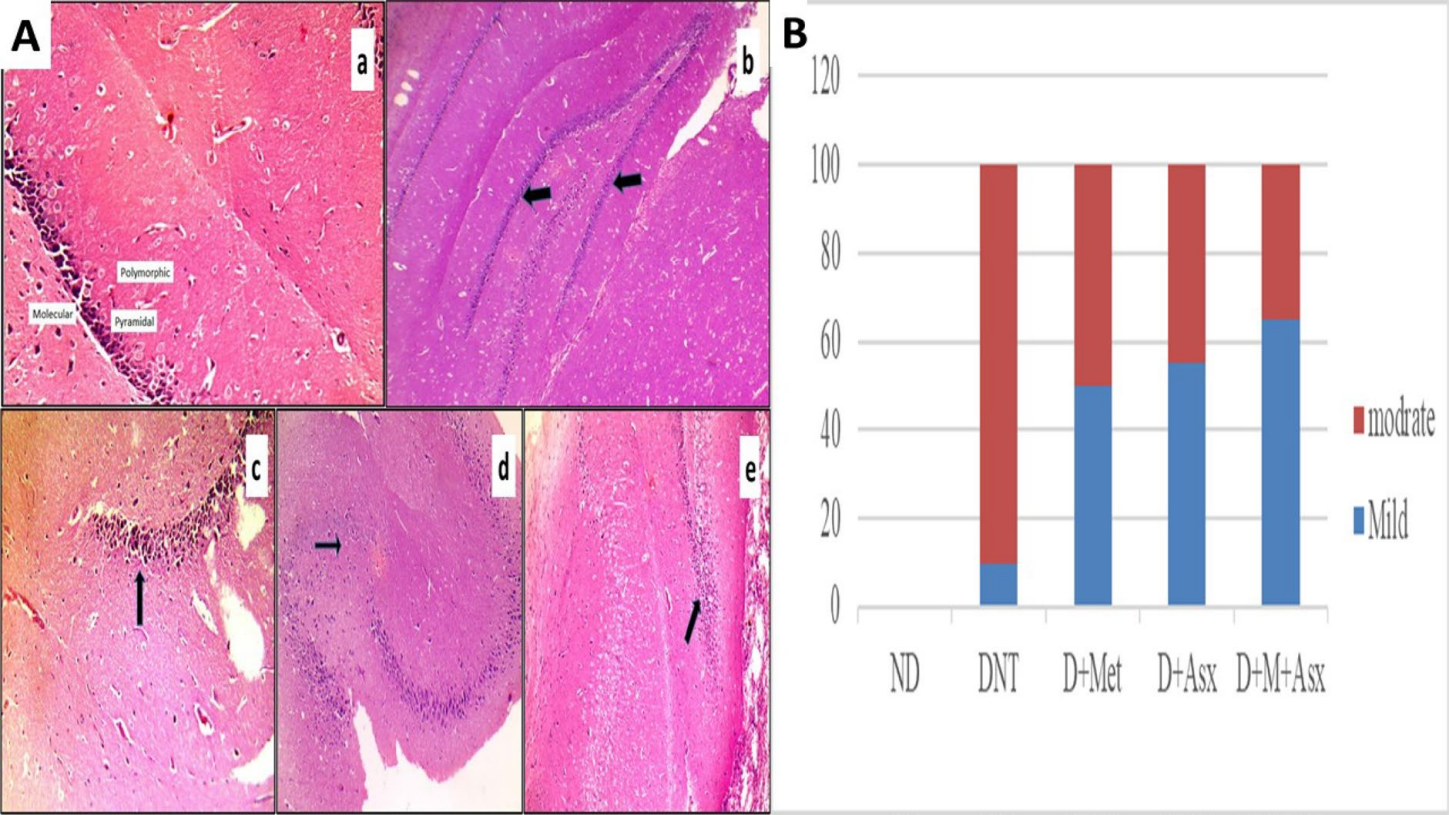
(**A**) a: ND group; hippocampus structure showed intact histological structure with pyramidal cell layer showing large rounded vesicular nuclei and prominent nucleoli (arrow), b: DNT group; hippocampus showing moderate degeneration (arrow), c: D + Met; hippocampus showing mild degeneration (arrow). d: D + Asx; hippocampus, showing mild degeneration (arrow), e: D + M + Asx; hippocampus, showing mild degeneration (arrow) (H&E original magnification X100). (**B**): degree of degeneration of hippocampus tissue.

Histopathological examination of routinely stained H & E slides showed moderate degeneration detected in hippocampus in 90% of DNT group and mild degeneration observed in 10% of the same group Fig. 4Ab,B, moderate degeneration detected in hippocampus in 50% of D + Met treated group and mild degeneration observed in 50% of the same group Fig. 4Ac,B, moderate degeneration detected in hippocampus in 45% of D + Asx treated group and mild degeneration observed in 55% of the same group Fig. 4Ad,B, and moderate degeneration detected in hippocampus in 35% of D + M + Asx treated group and mild degeneration observed in 65% of the same group Fig. 4Ae,B.

**Fig. 5:**
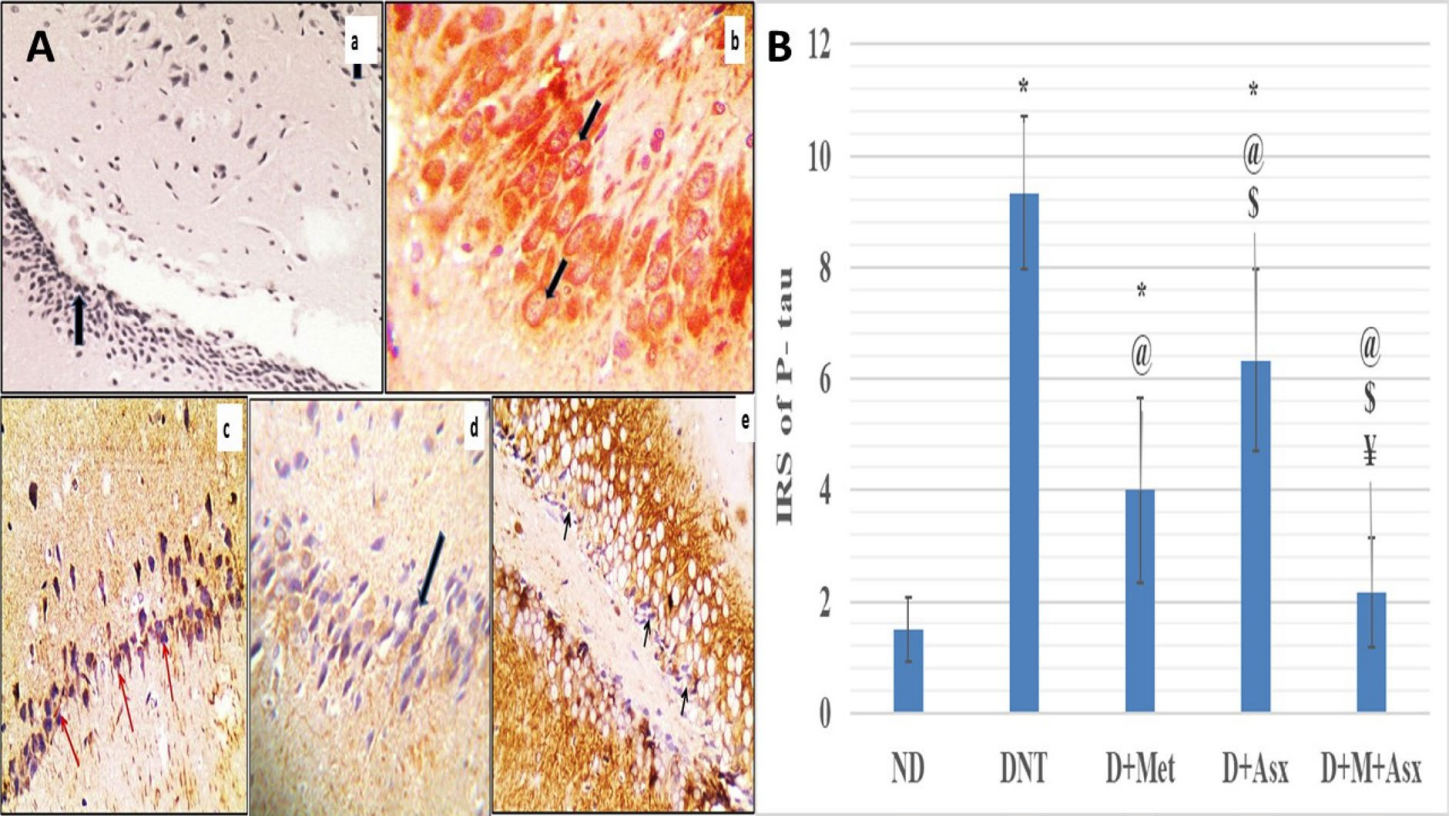
(**A**) a: ND group; hippocampus, showing negative phospho-tau expression (arrows), b: DNT group; showing strong cytoplasmic phospho-tau expression, c: D + Met group; hippocampus, showing mild phospho-tau expression (arrows) d: D + Asx group; hippocampus, showing mild phospho-tau expression (arrows). e: D + M + Asx group; hippocampus, showing mild phospho-tau expression (arrows). (IHC original magnification × 400). (**B**) IRS of P-tau of Hippocampus tissue in non-diabetic (ND), diabetic non-treated (DNT), diabetic metformin-treated (D + Met), diabetic astaxanthin-treated (D + Asx) and diabetic combined metformin & astaxanthin treated groups (D + M + Asx). [*significant when compared to ND group, #significant when compared to DNT group, $ significant when compared to (D + Met), ¥ significant when compared to(D + Asx)]. Data are shown as means + SD (n = 10). ANOVA was used to make group comparisons; Significance = *P* < 0.05). # No. of rats in each group was 18.

Immunohistochemistry “IHC” staining against phospho-tau showed that tau is expressed as brownish granular colour, the mean value of immunoreactive scoring “IRS” of p-tau of DNT group was significantly greater in comparison to ND group (Fig. 5Ab). The mean values of “IRS” of p-tau of D + Met and D + Asx treated groups were significantly lower when compared to DNT group (Fig. 5Ac,d), while remained significantly higher when compared to ND group. The mean value of IRS of p-tau of D + Asx treated group was notably more than D + Met treated group.

The mean value of “IRS” of p-tau of D + M + Asx treated group was significantly lesser than DNT group (Fig. 5Ab), D + Met and D + Asx treated groups, while showed insignificant change when compared to ND group (Fig. 5B).

There was a significant negative correlation between “TAC” and both “Hb A1C” and "HOMA-IR" (Table 5), there was also a significant negative correlation between “TAC” and “IRS” of p-tau, while there was a significant positive correlation between IRS of p-tau and the escape latency of the probe trial of MWM test, and a significant negative correlation between “IRS” of p-tau and % of spontaneous alternation of Y-maze, and also a significant negative correlation between “IRS” of p-tau and both d2 and d3 (Table 6).

**Table 5:** Correlation between TAC with HbA1c and HOMA-IR.

			TAC	HbA1C	HOMAIR
Spearman’s rho	TAC	Correlation Coefficient	1.000	− 0.512**	− 0.624**
		Sig. (2-tailed)		**0.005**	**0.001**
		N	30	28	27

**Correlation is significant at the 0.01 level (2-tailed).

*Correlation is significant at the 0.05 level (2-tailed).

Significant values are in [bold].

**Table 6:** Correlation between TAC with P-tau and cognitive brain functions.

			TAC	Ptau	Probe	Ymaze	d2	d3
Spearman’s rho	TAC	Correlation Coefficient	1.000	− 0.613**				
		Sig. (2-tailed)		**0.001**				
		N	30	28				
	Ptau	Correlation Coefficient	− 0.613**	10.000	0.376	− 0.406*	− 0.417*	− 0.416*
		Sig. (2-tailed)	0.001	0	**0.053**	**0.036**	**0.027**	**0.028**
		N	28	28	27	27	28	28

**Correlation is significant at the 0.01 level (2-tailed).

*Correlation is significant at the 0.05 level (2-tailed).

Significant values are in [bold].

“TAC” showed a significant positive correlation with intact DNA, significant negative correlation with maximal optical density of hippocampal RNA, significant negative correlation with maximal optical density of apoptotic fragments of DNA, significant negative correlation with the escape latency of the probe trial of “MWM” test, significant positive correlation with% of spontaneous alternations of Y-maze, and significant positive correlation with both d2 and d2(Table 7).

**Table 7:** Correlation between TAC with intact DNA, RNA and DNA fragmentations and cognitive brain functions.

			TAC	intactDNA	RNA	DNAfrags	probe	Ymaze	d2	d3
Spearman’s rho	TAC	Correlation Coefficient	1.000	0.484**	− 0.473**	− 0.785**	− 0.706**	0.367*	0.430*	0.430*
		Sig. (2-tailed)		**0.007**	**0.008**	**0.000**	0.000	0.050	0.018	0.018
		N	30	30	30	30	28	29	30	30
	intactDNA	Correlation Coefficient	0.484**	1.000	− 0.412*	− 0.679**	− 0.378*	0.440*	0.415*	0.419*
		Sig. (2-tailed)	0.007		0.024	0.000	**0.047**	**0.017**	**0.022**	**0.021**
		N	30	30	30	30	28	29	30	30
	RNA	Correlation Coefficient					0.093	0.171	− 0.231	− 0.229
		Sig. (2-tailed)					0.636	0.374	0.219	0.223
		N					28	29	30	30
	DNAfrags	Correlation Coefficient					0.456*	− 0.211	− 0.450*	− 0.450*
		Sig. (2-tailed)					**0.015**	**0.271**	**0.013**	**0.013**
		N					28	29	30	30

**Correlation is significant at the 0.01 level (2-tailed).

*Correlation is significant at the 0.05 level (2-tailed).

Significant values are in [bold].

Intact DNA showed significant negative correlations with maximal optical density of hippocampal RNA, maximal optical density of apoptotic fragments of DNA, and the escape latency of the probe trial of “MWM” test. While showed significant positive correlation with% of spontaneous alternations of Y-maze, and with both d2 and d2 (Table 7).

Maximal optical density of apoptotic fragments of DNA showed significant positive correlation with the escape latency of the probe trial of “MWM” test, significant negative correlation with% of spontaneous alternations of Y-maze, and with both d2 and d2 (Table 7).

## Discussion

In the present investigation, feeding rats with HFD for 2 weeks and a low dose of STZ (35 mg/kg) led to a significant rise in fasting serum glucose and HbA1c, associated with a significant decrease in serum insulin, when compared to the corresponding values in the non-diabetic group. The model was authenticated by calculating HOMA IR. The results showed a significant rise in HOMA IR in the diabetic non-treated group when compared to the non-diabetic group. A reduction in the overall number of insulin receptors without a change in receptor affinity can account for the insulin resistance brought on by HFD in the current study.^28^. In addition, HFD decreases insulin receptor autophosphorylation and IRS-1 phosphorylation^29^ as well as activation of phosphatidylinositol-3 kinase and protein kinase B phosphorylation and activity^30^.

The results of this group showed that cognitive brain functions, including spatial memory and learning, non-spatial memory, and working memory, investigated by escape latency (in seconds) of the Morris water maze test, discrimination index of the Novel Object Recognition test, and percentage of alterations of the Y maze test, respectively, of the diabetic non-treated group were significantly elevated, with the latter two being significantly reduced when compared to the corresponding values of the non-diabetic group. The decline of cognitive functions in this group can be explained by the significant increase of phosphorylated tau protein investigated by immunohistochemistry in the hippocampus tissue of the diabetic non-treated rats when compared with those in the non-diabetic group. Since tau protein stabilizes neuronal microtubules by attaching to them and promoting tubulin assembly into microtubules^31^.

There is growing evidence that tau hyperphosphorylation leads to axonopathy and contributes to the cognitive loss associated with diabetes mellitus^32^. Hyperphosphorylated tau affects microtubule structure and decreases microtubule stability^33^. The state of insulin resistance brought on by giving rats a low dosage of STZ (35 mg/kg) and a high-fat diet for 2 weeks can account for the increase in phosphorylated tau protein. Additionally, there was a strong positive association between the HOMA IR score and the increase of the phosphorylated tau protein.

Although it is widely accepted that several protein kinases control tau phosphorylation, GSK-3β is the most significant tau kinase and is primarily dynamically controlled by the insulin signalling system^32,33^. The aberrant insulin signalling pathway, which involves insulin and IGF-1, is unable to start downstream signalling because IRS-1 and IRS-2 are more hyperphosphorylated while IR and IGF-1 R are less phosphorylated. Akt inhibition activates GSK3β by reducing its phosphorylation at serine 9. Activated GSK3β then causes abnormal tau phosphorylation and neuronal death^34,35^.

Because inflammation is linked to decreased insulin sensitivity, it is assumed to be a significant factor in the development of type 2 diabetes mellitus. TNF-α and IL-6 may cause insulin resistance by suppressing the expression of GLUT-4 and insulin receptor substrate-1 (IRS-1) and activating the NF-κβ pathway^36^. In our investigation, the fasting serum level of Il-6 was significantly higher in the DNT group when compared to the ND group.

Since high plasma cholesterol at midlife is linked to greater Aβ40 levels^37^ and a 2–threefold increased risk for later Alzheimer’s disease dementia^38^, dyslipidaemia may be an additional mechanism linking the pathophysiology of type 2 DM. In our investigation, the results of the DNT group showed significantly higher TC, TG, and LDL-c and a significantly lower HDL-c when compared to the corresponding values of the ND group.

A substantial amount of data points to insulin resistance as a key factor in the development of dyslipidaemia^39^. Tyrosine kinase activity, receptor phosphorylation, and IRS phosphorylation all decrease in muscle and adipocytes when insulin binds to its receptor. Moreover, tissue-specific alterations exist: IRS-2 becomes the main PI3K docking protein when IRS-1-associated PI3K activity declines due to decreased IRS-1 expression in the adipocytes of obese individuals with type 2 diabetes^40^. On the other hand, IRS-1 and IRS-2 protein levels are normal in the skeletal muscle of obese, type 2 diabetes people, but PI3K activity linked to both IRSs is compromised^41^.

At the molecular level, the DNT group showed significant DNA changes in the form of significantly increased optical density of electrophoretic hippocampal RNA, significantly decreased optical density of intact hippocampal DNA, and an apoptotic pattern of DNA fragmentation affecting hippocampal tissue when compared to the corresponding values of the ND group. This decline in cognitive function was proved by histopathological studies; sections showed moderate degeneration was detected in the hippocampus in 90% of the DNT group and mild degeneration was observed in 10% of the same group.

There is strong evidence that insulin regulates the production of the DNA repair enzyme XPD, which is essential for nucleotide excision repair^42^. Additionally, those investigations showed that extended exposure to high glucose concentrations may increase the degree of DNA damage and downregulate the insulin-dependent increase in XPD mRNA levels. The results of these studies also imply that^43^ the degree of insulin resistance should be considered when discussing DNA damage and repair in diabetes. In our investigation, a statistically significant increase in fasting serum glucose and HbA1c and a statistically significant decrease in fasting serum insulin levels could explain these DNA changes.

According to Nishida et al.^44^, DNA is susceptible to lesions associated to oxidative stress, the most characteristic of which is the creation of 8-oxo-2′-deoxyguanosine (8-OHdG). This lesion is highly harmful and may cause both mutagenesis and apoptosis.

Because hypoinsulinemia increases the activity of the fatty acyl coenzyme, it can help explain the oxidative stress associated with diabetes. When an enzyme initiates the oxidation of fatty acids, it increases lipid peroxidation, which impairs membrane function by decreasing membrane fluidity and changing the activity of membrane-bound enzymes and receptors. The results of lipid peroxidation harm most of the body’s cells and are associated with several diseases, such as brain damage and atherosclerosis^45^.

According to the hypothesis, Asx increases the autophosphorylation of insulin receptor-β (IR-β), IRS-1associated PI3-kinase step, phospho-Akt/Akt ratio, and GLUT-4 translocation in skeletal muscles, thereby intensifying post-receptor signalling events^46^. By controlling the activation of 5′ adenosine monophosphate-activated protein kinase (AMPK) in the muscle, Asx treatment markedly reduced insulin resistance and glucose intolerance. Furthermore, it promoted the biogenesis of mitochondria in muscles^47^.

As an antidiabetic medication, metformin is regarded as an insulin sensitizer as it normalizes blood sugar levels without promoting the release of insulin^48^. This is due to the fact that increasing the phosphorylation state of AMPK, an essential controller of energy balance in cells and the body, activates it without changing the AMP/ATP ratio^49,50^. It turns out that GLUT-4 translocation to the membrane, enhanced glucose uptake in the muscles and liver, glycolysis, fatty acid oxidation, and reduced gluconeogenesis, glycogen, fatty acid, and cholesterol production are all associated with higher AMPK activity^51,52^. Additionally, AMPK causes acetyl-CoA carboxylase (ACC) to become phosphorylated and inactive, which lowers triglyceride levels. This drop can be accompanied by either a decrease in fatty acid synthesis or an increase in fatty acid oxidation^53^.

The inhibition of gluconeogenesis seen in type 2 diabetic patients treated with metformin may be explained by the drug’s inhibition of the target of rapamycin (TOR) pathway through AMPK activation^54^. Additionally, by blocking the TOR pathway, AMPK prevents protein synthesis in a variety of cells^55^.

This could explain the significant improvement in the performance of behavioural tasks in MWM, y-maze, and NOR when compared to the DNT, D + Met, and D + Asx groups.

At the molecular level, the combined Met and Asx diabetic treated group showed significant DNA changes in the form of significantly increased optical density of electrophoretic hippocampal RNA, significantly increased optical density of intact hippocampal DNA, and the apoptotic pattern of DNA fragmentation affecting hippocampal tissue when compared to the corresponding values of DNT, D + Met, and D + Asx groups. This improvement was spotted at the cellular level by histopathological examination of hippocampus tissue in diabetic combined metformin & astaxanthin-treated group by H&E stain, which showed mild to moderate degeneration in a ratio of 1.85:1 affecting hippocampus tissue.

The present investigation demonstrated that the combination of Astaxanthine (1 mg/kg) and MET (200 mg/kg) for 4 weeks revealed a statistically significant decrease in fasting serum glucose, HbA1c, HOMA-IR, TC, TG, LDL-c, MDA, and IL-6, while HDL-c and TAC were significantly higher when compared to the corresponding values of DNT, D + Met, and D + Asx groups. Moreover, the IRS of phosphorylated tau protein was significantly decreased in the hippocampal tissues when compared to the corresponding values in the DNT, D + Met, and D + Asx groups.

The improvement in all previously investigated parameters can be explained on the basis of the combination of hypoglycaemic, antioxidant, and anti-inflammatory effects of metformin, in addition to the hypoglycaemic, antioxidant, and anti-inflammatory effects of astaxanthin.

## Conclusion

It is possible to come to the following conclusions from the current study’s findings: Treatment with astaxanthine improves the glycaemic state by lowering fasting blood glucose and glycosylated haemoglobin, lowering insulin resistance, and enhancing cognitive brain functioning in diabetic rats. The glycaemic status and cognitive brain functions of diabetic rats were significantly improved by the combination of astaxanthine and metformin therapy. In type II diabetes mellitus, this improvement was greater than that of using either medication alone, which may suggest that astaxanthine had a supplemental impact to metformin therapy. One possible adjuvant treatment for type II diabetes mellitus is astaxanthine. We suggest further studies to explore other pathways to provide a more comprehensive understanding of astaxanthin’s multifaceted mechanisms of action.

## Data Availability

The datasets used and/or analyzed in the current investigation are accessible from the corresponding author up on reasonable request.
